# Managing cognitive impairment in patients with chronic obstructive pulmonary disease (COPD) in Saudi Arabia: what are the current practices?

**DOI:** 10.1080/07853890.2024.2413924

**Published:** 2025-01-28

**Authors:** Rayan A. Siraj, Ahmed M. Alrajeh, Mushabbab A. Alahmari, Fahad H. Alahmadi, Abdulelah M. Aldhahir, Abdullah A. Alqarni, Jaber S. Alqahtani, Saeed M. Alghamdi, Turki M. Alanazi, Abdullah Alruwaili, Saleh S. Algarni, Abdulrhman S. Alghamdi, Tawah H. Alsindi, Mohammed M. Alyami, Faisal A. Alshehri, Talal Alshammari, Rehab Alhasani, Khalid Alrashed, Nawaf E. Sabir, Ataa F. Mesbah

**Affiliations:** a Department of Respiratory Care, College of Applied Medical Sciences, King Faisal University, Hofuf, Saudi Arabia; b Department of Respiratory Therapy, College of Applied Medical Sciences, University of Bisha, Bisha, Saudi Arabia; c Health and Humanities Research Center, University of Bisha, Bisha, Saudi Arabia; d Respiratory Therapy Department, College of Medical Rehabilitation Sciences, Taibah University, Madinah, Saudi Arabia; e Respiratory Therapy Program, Nursing Department, College of Nursing and Health Sciences, Jazan University, Jazan, Saudi Arabia; f Department of Respiratory Therapy, Faculty of Medical Rehabilitation Sciences, King Abdulaziz University, Jeddah, Saudi Arabia; g Respiratory Therapy Unit, King Abdulaziz University Hospital, Jeddah, Saudi Arabia; h Department of Respiratory Care, Prince Sultan Military College of Health Sciences, Dammam, Saudi Arabia; i Clinical Technology Department, Respiratory Care Program, Faculty of Applied Medical Sciences, Umm Al-Qura University, Makkah, Saudi Arabia; j Department of Respiratory Therapy, King Saud Bin Abdelaziz University for Health Sciences, Al Ahsa, Saudi Arabia; k King Abdullah International Medical Research Center, Al Ahsa, Saudi Arabia; l Emergency Medical Services program, College of Applied Medical Sciences, King Saud bin Abdulaziz University for Health Sciences, Al Ahsa, Saudi Arabia; m Department of Respiratory Therapy, College of Applied Medical Sciences, King Saud bin Abdulaziz University for Health Sciences, Riyadh, Saudi Arabia; n King Abdullah International Medical Research Center, Riyadh, Saudi Arabia; o Department of Rehabilitation Sciences, College of Applied Medical Sciences, King Saud University, Riyadh, Saudi Arabia; p Department of Respiratory Therapy Program, Inaya Medical College, Riyadh, Saudi Arabia; q Respiratory Therapy Department, Batterjee Medical College, Khamis Mushait, Saudi Arabia; r Respiratory Therapy Program, Batterjee Medical College, Jeddah, Saudi Arabia; s Occupational Therapy Department, College of Applied Medical Sciences, King Saud bin Abdulaziz University for Health Sciences, Al Ahsa, Saudi Arabia; t Department of Rehabilitation Sciences, College of Health and Rehabilitation Sciences, Princess Nourah bint Abdulrahman University, Riyadh, Saudi Arabia; u Neurology Department, King Fahad Specialist Hospital- Dammam, Dammam, Saudi Arabia; v Adult Critical Care Department, King Abdulaziz University Hospital-Jeddah, Jeddah, Saudi Arabia

**Keywords:** Cognitive dysfunction, comorbidity, dementia, pulmonary disease, chronic obstructive

## Abstract

**Objective:**

Cognitive impairment is a common comorbidity, yet overlooked, in patients with chronic obstructive pulmonary disease (COPD). However, little is known about the current practice and perceptions of physicians on recognising and managing cognitive impairment in patients with COPD in Saudi Arabia. This study aimed to investigate current practices and perceptions of physicians in Saudi Arabia regarding the recognition and management of cognitive impairment in COPD patients.

**Methods:**

An online cross-sectional questionnaire was distributed between March and October 2023 to physicians in Saudi Arabia. The collected responses were analysed using descriptive statistics.

**Results:**

A total of 808 physicians completed the online survey. Of whom, only 19% indicated receiving adequate training for managing cognitive impairment. Although the vast majority of physicians reported that cognitive impairment leads to underestimation of COPD severity (85%) and interferes with self-management (85%), only 11% agreed on the important role of screening. In addition, only half of the study participants aimed to identify possible cognitive impairment, with only 4% screening for cognitive impairment during patients’ assessment. The overall confidence level in recognising and managing cognitive impairment was relatively low. The most common barriers contributing to the suboptimal management of cognitive impairment in COPD were poor training (62%), the absence of standardised procedures (63%) and limited knowledge (58%) about cognitive impairment in COPD.

**Conclusion:**

The current practice of recognising and managing cognitive impairment in Saudi Arabia is suboptimal. This is likely to be attributed to inadequate training, the absence of standardised procedures, and limited knowledge about cognitive impairment in COPD. Healthcare systems should provide more training and implement a holistic approach to detect and manage cognitive impairment during patients’ visits.

## Introduction

Chronic obstructive pulmonary disease (COPD) is frequently accompanied by comorbidities, which have substantial impacts on clinical management, one of which is cognitive impairment [1]. Indeed, the presence of cognitive impairment in patients with COPD may result in an increased need for healthcare utilisations [2], inadequate adherence to medication [3], increased functional dependence [4], improper inhaler usage [4], and decreased completion rates of pulmonary rehabilitation programs [5]; thus, leading to an elevated risk of hospitalisation and even mortality in some cases [6]. Because of this, it is crucial to recognise cognitive impairment [1,7] in patients with COPD and implement appropriate interventions to address this issue.

Current estimates of cognitive impairment in patients with COPD range from 4% to 61% [8,9], and that 1 in 4 patients with COPD suffers from cognitive impairment [10]. The current prevalence variability is mainly attributed to demographic differences such as sample size, study designs, and methods used to assess cognitive impairments [1]. A comprehensive population-based study conducted in the United Kingdom revealed that individuals with COPD are at a 13% higher risk of developing cognitive impairment and subsequent dementia compared to matched subjects without COPD [11]. While no studies have been conducted to estimate the prevalence and incidence of cognitive impairment in COPD in the Saudi Arabian population, current data show that the prevalence and incidence of COPD have been significantly rising over the past decades (1990-2019) [12], hence increasing the prevalence of associated comorbidities such as cognitive impairment. Despite the recommendations of international guidelines, such as Global Initiatives for Chronic Lung Disease [13], to identify and manage comorbidities (e.g. cognitive impairment) in COPD patients, cognitive impairment is not commonly assessed during COPD visits [13]. Current COPD guidelines suggest that comorbidities in COPD, one of which is cognitive impairment, should be recognised and managed according to usual care (as if the patient does not have COPD). This, in other words, may imply that cognitive impairment symptoms should be screened for patients with COPD who undergo physical assessment [13].

An efficacious clinical approach to the management of cognitive impairment in individuals with COPD commences with the prompt identification of this condition [14]. Undetected cognitive impairment symptoms, such as impaired memory, language, motor function, and attention, may increase subrationally, contributing to increased dependence and decreased social interaction in patients with COPD [15]. Failure to diagnose or underdiagnose cognitive impairment in COPD patients may result in delayed intervention, thereby exacerbating the impact of cognitive impairment and potentially hindering the prevention of irreversible dementia [16], as cognitive impairment serves as a precursor to established dementia [17,18]. In fact, evidence suggests that 30% of patients with cognitive impairment (excluding COPD) may develop dementia within a median period of 5 years [16]. Consequently, early recognition of cognitive impairment is of significant clinical importance in facilitating timely and appropriate interventions.

Healthcare professionals play a crucial role in the identification of cognitive impairment during patient encounters, enabling them to deliver optimal healthcare services. However, there is limited data available on the knowledge and current practice of recognising and managing cognitive impairment in patients with COPD. Thus, this study aimed to 1) investigate the attitudes of physicians on recognising and managing cognitive impairment in patients with COPD, 2) assess the current practice and confidence level, and 3) explore the barriers and facilitators interfering with optimal practice.

## Methods

### Study design and participants

This study utilised an online survey – distributed through an electronic platform (Survey Monkey) - to evaluate physicians’ attitudes, current practices, and challenges related to managing cognitive impairment in patients with COPD. The survey was conducted from March to October 2023.

### Questionnaire tool

The questionnaire used in this study comprised five distinct sections with closed-ended questions. English was used when applying the questionnaire, as it is the primary language used within the Saudi healthcare system. This study has adopted a previously validated questionnaire, which has been used to assess the management of a common comorbidity (e.g. depression) in patients with COPD and Asthma [19,20]. Formulations of the questions were based on the current literature on cognitive impairment in patients with COPD [1,7,15]. This is done to ensure alignment with the specific aims of this study. A multidisciplinary panel of experts, including specialists in respiratory medicine, respiratory epidemiology, physical and occupational therapy, and neurology, meticulously reformulated the original questionnaires. The final version of the questionnaire was validated through pilot testing of 20 physicians from various specialities, such as general practitioners (GPs), internal medicine, family medicine, and pulmonary medicine. Notably, participants involved in the pilot testing phase were excluded from the primary study. The structured format of the questionnaire was as follows:

Section 1: Participant background information was collected, including demographic data such as sex, current occupation, clinical experience (in years), average patient interaction time, monthly caseload of COPD patients, and whether physicians had received previous training in cognitive impairment management in people with COPD.
Section 2: Physicians’ perspectives on cognitive impairment in COPD patients were assessed using a 5-point Likert scale ranging from 1 (strongly disagreed) to 5 (strongly agreed). Ten statements were presented to gauge their agreement or disagreement with each statement. At the end of Section 2, participants were asked whether they aimed to identify possible cognitive impairment as part of patient management for patients with COPD. Those who answered ‘No’ were skipped from Sections 3 and 4 and moved directly to Section 5 (assessing barriers and facilitators).
Section 3: Aimed to explore physicians’ current practices when working with patients with COPD and cognitive impairment. This section consists of three specific questions: 1) The extent to which physicians desire to identify cognitive impairment in individuals with COPD, 2) The utilisation of screening tools for cognitive impairment, and 3) The likelihood of taking appropriate actions upon assuming cognitive impairment in COPD patients.
Section 4: Assesses Physicians’ confidence levels in managing cognitive impairment in COPD patients using a 5-point Likert scale ranging from 1 (not confident) to 5 (completely confident). Nine statements were provided to measure the level of confidence.
Section 5: Two questions were included to assess physicians’ perceptions of the facilitators and barriers to diagnosing cognitive impairment in COPD patients. These questions aimed to identify factors that either support or hinder the process of diagnosing depression in this specific patient population.

### Participants and sampling approach

Participant recruitment was done through a convenience sampling technique. Physicians who are more likely to conduct a standard assessment for COPD patients and follow their physical illness, such as GPs, family medicine practitioners, internal medicine physicians, and pulmonologists/respirologists, were included. Physicians must have an active licence, practice in Saudi Arabia, and consent to participate in the study.

To ensure representation from all geographical areas in Saudi Arabia, authors from multiple institutions in various regions participated in the data collection process. Professional Saudi committees, such as the Saudi Society of Internal Medicine, the Saudi Thoracic Society, the Saudi Society for Respiratory Care, and the Saudi Society of Family and Community Medicine, were approached to distribute the survey among physicians. Contributing authors also participated in the data collection, each assigned to a specific area in the country and followed up with hospital officials to recruit the targeted participants and cover all Saudi regions. The survey was also sent through social media networks (X, WhatsApp, and Telegram) to maximise participation. Each author was assigned to a specific country area to ensure comprehensive coverage.

Prior to the study, participants were informed about the purpose and identity of the principal investigator. They were assured that participation was voluntary and their data would be treated confidentially. It was also made clear that no personal information would be shared and that it would be deleted as soon as it was processed. The survey was designed to be completed within approximately 5-7 min. Participants were encouraged to submit a complete survey; incomplete ones were excluded.

### Sample size

The Saudi healthcare system is overseen and structured by the Saudi Health Council. This governing body ensures that regulations are consistent across different healthcare organizations. A government report from 2023 indicates that there are a total of 35,978 physicians in specific categories: 15,856 general practitioners, 727 pulmonologists, 10,242 family medicine practitioners, and 9,153 internal medicine specialists [21]. The sample size calculation used the formula for estimating proportions to determine the proportion of physicians who aim to identify cognitive impairment in patients with COPD [22,23]. Since no previous data is available, a conservative estimate of 50% was used. The calculation was then adjusted using the finite population correction due to the finite size of the study population (*N* = 35,978), indicating that a sample size of 652 participants is required to achieve a 99% confidence level with a 5% margin of error [22,23].

### Ethical consideration

Ethical approval for the study was obtained from an independent research committee at King Faisal University (ID: KFU-REC-2022-NOV-ETHICS291). Written consent forms were obtained from participants prior to the start of filling out the questionnaire. The following question was asked: ‘Do you agree to take part in the study?’. If participants ticked ‘yes’, they have given their consent to take part in the survey. The study was conducted in accordance with the principles of the Declaration of Helsinki.

### Statistical analysis

The statistical analyses, including data management, were conducted using STATA version 16.0 software (StataCorp LP, College Station, TX, US). As all variables in this study were categorical, the results were reported and presented as numbers and percentages. Missing or incomplete questionnaires were excluded from the study results. In addition, the relationship between demographic variables (age, sex, geographical locations, place of work, and years of experience) and having received training for managing cognitive impairment in COPD were assessed using logistic regression. The Odds (OR) with a 95% confidence interval (CI) were calculated using bivariate logistic regression models, with each demographic variable included in a separate model.

## Results

Between March and October 2023, a total of 808 physicians from different specialities across Saudi Arabia (Figure S1) completed the online survey, of whom 75.74% were male, Table 1. Most respondents were aged 31-40 years old (43.40%), with 1 - 4 years of experience (43.19%). Internal medicine doctors accounted for 36.51% of the total participants, followed by family medicine, GPs, and pulmonary/respiratory medicine physicians, 34.03%, 22.03%, and 7.4%, respectively. Disappointingly, the vast majority of participants did not receive adequate training on managing cognitive impairment (80%). The respondents’ demographic and experience information is summarised in Table 1.

**Table 1.** Demographics of the study respondents (*n* = 808).

Demographics	Frequency (%)
Sex	
Mle	612 (75.74%)
Female	196 (24.26%)
Age	
24-30 years old	254 (31.39%)
31-40 years old	351 (43.40%)
41-50 years old	154 (19.14%)
>50 years old	49 (6.07%)
Geographic Location	
Central Region	212 (26.24%)
Eastern Region	132 (16.34%)
Western Region	80 (9.90%)
Northern Region	84 (10.40 %)
Southern Region	300 (37.13 %)
Profession	
Internal medicine	295 (36.51%)
Family medicine	275 (34.03%)
General Practitioner	178 (22.03%)
Pulmonologist	60 (7.43%)
Primary place of work	
Governmental hospital	643 (79.58%)
Private hospital	165 (20.42%)
The average number of COPD patients seen per month	
≤10	247 (30.75%)
11–20	358 (44.31)
≥21	203 (25.57%)
Years of clinical experience with COPD patients	
<1 year	38 (4.70%)
1–4 years	349 (43.19%)
5–9 years	278 (34.41%)
≥10 years	143 (17.70%)
Average time spent with each COPD patient	
≤10 min	203 (25.12%)
11–20 min	296 (36.63%)
21–30 min	145 (17.95%)
31–40 min	102 (12.62%)
41–50 min	53 (6.56%)
51–60 min	6 (0.74%)
≥60 min	3 (0.37%)
Received specific training for managing cognitive impairment	
Yes	148 (18.32%)
No	660 (81.68%)

Data are presented as frequency and percentage unless stated otherwise.

### Physicians’ views about cognitive impairment in patients with COPD

The majority of the 808 respondents who completed the survey either agree (34.16%) or strongly agree (40.10%) that cognitive impairment may develop due to impaired lung function, such as COPD, Table 2. Furthermore, more than 80% of the participants indicated that cognitive impairment might lead to either COPD underdiagnoses or misdiagnosis of COPD and is also responsible for poor adherence to medications and insufficient adherence to pulmonary rehabilitation in patients with COPD. In addition, 77% of the participants agreed that cognitive impairment may lead to underestimation of exercise performance and treatment refusal in patients with COPD.

**Table 2.** Physicians’ views about cognitive impairment in patients with COPD (*n* = 808).

Item	Frequency (%)
Cognitive impairment may develop as a result of impaired lung function, such as COPD	
Strongly disagree	7 (0.87%)
Disagree	13 (1.61%)
Neither agree nor disagree	188 (23.27%)
Agree	276 (34.16%)
Strongly agree	324 (40.10%)
Cognitive impairment may lead to underdiagnoses of COPD	
Strongly disagree	3 (0.37%)
Disagree	8 (0.99%)
Neither agree nor disagree	79 (9.78%)
Agree	337 (41.71%)
Strongly agree	381 (47.15%)
Cognitive impairment may lead to a misdiagnosis of the COPD severity	
Strongly disagree	1 (0.12%)
Disagree	5 (0.62%)
Neither agree nor disagree	108 (13.37%)
Agree	256 (31.68%)
Strongly agree	438 (54.21%)
Cognitive impairment is responsible for poor adherence to medications in patients with COPD	
Strongly disagree	1 (0.12%)
Disagree	11 (1.36%)
Neither agree nor disagree	114 (14.11%)
Agree	288 (35.64%)
Strongly agree	394 (48.76%)
Cognitive impairment is responsible for insufficient adherence to pulmonary rehabilitation in patients with COPD.	
Strongly disagree	1 (0.12%)
Disagree	10 (1.24%)
Neither agree nor disagree	102 (12.62%)
Agree	327 (40.47%)
Strongly agree	368 (45.54%)
Cognitive impairment may lead to underestimation of exercise performance in patients with COPD	
Strongly disagree	28 (3.74%)
Disagree	6 (0.74%)
Neither agree nor disagree	145 (17.95%)
Agree	269 (33.29%)
Strongly agree	360 (44.55%)
Cognitive impairment may lead to treatment refusal in patients with COPD	
Strongly disagree	2 (0.25%)
Disagree	5 (0.62%)
Neither agree nor disagree	105 (13.00%)
Agree	259 (32.05%)
Strongly agree	437 (54.08%)
There is little value in routinely screening for cognitive impairment in patients with COPD.	
Strongly disagree	3 (0.37%)
Disagree	10 (10.24%)
Neither agree nor disagree	96 (11.88%)
Agree	302 (37.37%)
Strongly agree	397 (49.13%)
Cognitive impairment exacerbates the symptoms of COPD.	
Strongly disagree	3 (0.37%)
Disagree	9 (1.11%)
Neither agree nor disagree	110 (13.61%)
Agree	274 (33.91%)
Strongly agree	412 (50.99%)
Cognitive impairment impairs patient self-management of COPD	
Strongly disagree	1 (0.12%)
Disagree	6 (0.74%)
Neither agree nor disagree	114 (14.10%)
Agree	272 (33.66%)
Strongly agree	415 (51.36%)
Do you aim to identify possible cognitive impairment as part of patient management for patients with COPD?	
Yes	415 (51.63%)
No	393 (48.63%)

Data are presented as frequency and percentage unless stated otherwise.

While the vast majority of respondents agreed on the impact of cognitive impairment on self-management in patients with COPD, they nevertheless see little value in conducting routine cognitive impairment screening in COPD patients. Of significant importance, though, is that only 51% of the study participants have the intention to identify possible cognitive impairment as part of management for patients with COPD, while the others said they do not aim to identify potential impairment.

### Current practices and confidence level for managing cognitive impairment in patients with COPD (n = 415)

Upon assessing the current practices of managing cognitive impairment in COPD, the majority of respondents (315 out of 415 (76%) participants who completed Sections 3 and 4 of the questionnaire) indicated that they either rarely or never used cognitive impairment screening tools in patients with COPD, Table 3. However, most physicians would ask patients about cognitive impairment symptoms during assessment and whether the patient has a family history of Alzheimer’s disease. None of the physicians indicated using formal diagnostic tools to confirm a diagnosis.

**Table 3.** Current practice in working with COPD patients (*n* = 415).

Item	Frequency (%)
Frequency of using cognitive impairment screening tool	
Always	2 (0.48%)
Often	13 (3.13%)
Sometimes	85 (20.48%)
Rarely	158 (38.07%)
Never	157 (37.83%)
When aiming to detect cognitive impairment in patients with COPD, what actions are you more likely to take?	
I take memory deficit as a criterion for cognitive impairment detection	240 (60.69%)
I take psychiatric symptoms as the criteria for cognitive impairment detection	352 (84.67%)
I would ask if the patient has a family history of Alzheimer’s disease	312 (75.77%)
I would detect risk factors	183 (44.75%)
I would discuss a probable diagnosis with the patient	260 (60.57%)
I would discuss a probable diagnosis with the family	248 (60.82%)
I would use a formal diagnostic questionnaire to confirm the diagnosis	0 (0 %)
When aiming to manage cognitive impairment in patients with COPD, what actions are you more likely to take?	
I would provide information about cognitive impairment	360 (86.33%)
I would provide a referral to a mental health clinic	333 (79.85%)
I would provide a referral to pulmonary rehabilitation	313 (75.05%)
I would provide medications	144 (34.53%)
I would provide supplemental oxygen therapy	276 (66.18%)

Data are presented as frequency and percentage unless stated otherwise.

### Demographic factors linked to receiving adequate training for managing cognitive impairment in patients with COPD

Logistic regression analysis assessed which demographic factors were linked to receiving adequate training for managing cognitive impairment in COPD patients. Results showed that male and female physicians do not differ in their training for managing cognitive impairment in patients with COPD (OR: 1.47; 95% CI: 0.99 − 2.18), Table 4. In addition, pulmonologists received significantly more training than GPs (OR: 3.75; 95% CI: 1.44 to 9.97). Further, middle-aged (31-40 years old and 41-50 years old) and older physicians (50 years and older) tend to be more adequately trained in managing cognitive impairment than those in their early careers. Similarly, physicians with more years of experience (≥ 10 years) are 4 times more likely to be trained in managing cognitive impairment in patients with COPD than those with less than 1 year of experience (OR: 4.23; 95% CI: 1.91 − 7.33; *p* < 0.001), Table 4.

**Table 4.** Bivariate logistic regression models of the factors associated with receiving specific training for managing cognitive impairment in patients with COPD.

Descriptor	OR (95% CI)
Sex	
Female	1
Male	1.47 (0.99 to 2.18)
Physician speciality	
GP	1
Family medicine	1.96 (0.89 to 4.28)
Internal medicine	1.73 (0.97 to 3.81)
Pulmonologist	3.75 (1.44 to 9.97)
Age group	
24–30 years	1
31–40 years	2.80 (1.77 to 4.44)
41–50 years	2.62 (1.55 to 4.43)
>50 years	1.67 (1.90 to 3.01)
Geographical location	
Central Region	1
Eastern Region	0.47 (0.26 to 0.85)
Northern Region	0.24 (0.13 to 0.44)
Southern Region	1.04 (0.60 to 1.81)
Western Region	0.22 (0.12 to 0.41)
Years of experience	
≤1 year	1
1–4 years	2.89 (1.44 to 5.80)
5–9 years	4.20 (2.03 to 8.67)
≥10 years	4.23 (1.91 to 7.33)
Place of work	
Governmental Hospital	1
Private Hospital	0.35 (0.23 to 0.52)

CI: confidence interval; GP: general practitioner; OR: odds ratio.

The level of confidence in working with patients with COPD and cognitive impairment was investigated by physicians (*n* = 415) who aimed to identify cognitive impairment during patients’ visits. The majority of respondents felt either slightly confident or not confident in all domains, such as responding to coexisting behavior problems; diagnosis and management; communicating the diagnosis to patients; discussing concerns with a patient’s family members, and disclosing a diagnosis to the patient or family member, Table 5.

**Table 5.** Confidence in working with patients with COPD and cognitive impairment (*n* = 415).

Item	Frequency (%)
In responding to co-existing behaviour problems in patients with COPD, I feel	
Completely confident	4 (0.96%)
Fairly confident	4 (0.96%)
Somewhat confident	113 (27.23%)
Slightly confident	196 (47.23%)
Not confident	98 (23.61%)
In telling the patients the diagnosis, I feel	
Completely confident	7 (1.69%)
Fairly confident	1 (0.24%)
Somewhat confident	41 (9.88%)
Slightly confident	212 (51.08%)
Not confident	154 (37.11%)
In discussing concerns about possible cognitive impairment with a patient’s family members, I feel	
Completely confident	51 (12.29%)
Fairly confident	8 (1.93%)
Somewhat confident	0 (0%)
Slightly confident	235 (56.63%)
Not confident	121 (29.16%)
In knowing which signs to look for to tell if a patient with COPD might be cognitively impaired, I feel	
Completely confident	6 (1.45%)
Fairly confident	1 (0.24%)
Somewhat confident	50 (12.05%)
Slightly confident	221 (53.25%)
Not confident	137 (33.01%)
In establishing a diagnosis of cognitive impairment, I feel	
Completely confident	62 (14.94%)
Fairly confident	9 (2.17%)
Somewhat confident	100 (24%)
Slightly confident	125 (30%)
Not confident	119 (28.67%)
In responding to co-existing psychiatric problems, I feel	
Completely confident	61 (14.70%)
Fairly confident	5 (1.20%)
Somewhat confident	96 (23.13%)
Slightly confident	138 (33.25%)
Not confident	115 (27.71%)
In providing education on the link between COPD and cognitive impairment, I feel	
Completely confident	6 (1.45%)
Fairly confident	2 (0.48%)
Somewhat confident	62 (14.945%)
Slightly confident	228 (54.94%)
Not confident	117 (28.19%)
In directing a patient who might be cognitively impaired to appropriate services or agencies, I feel	
Completely confident	9 (2.17%)
Fairly confident	2 (0.48%)
Somewhat confident	67 (16.14%)
Slightly confident	213 (51.33%)
Not confident	124 (29.88%)

Data are presented as frequency and percentage unless stated otherwise.

### Barriers to identifying and managing cognitive impairment in patients with COPD

**Figure 1.**
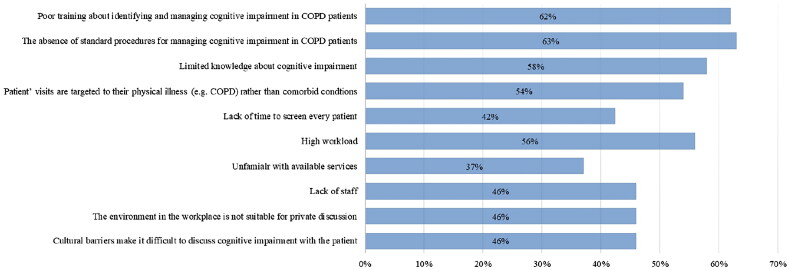
The most common barriers to identifying and managing cognitive impairment in patients with COPD (*n* = 808).

According to the respondents, the most common barriers to identifying and managing cognitive impairment in patients with COPD were poor training (62%), limited knowledge (58%), absence of standard procedures (63%), and lack of time to screen every patient (63%). The other barriers included High workload (56%), lack of team staff (46%), and the patients’ emphasis on physical illness rather than mental well-being (54%), Figure 1.

### Factors that facilitate the identification and management of cognitive impairment in patients with COPD

**Figure 2.**
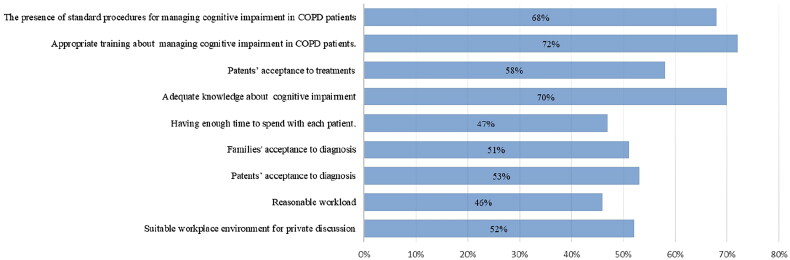
The most common factors that facilitate identifying and managing cognitive impairment in patients with COPD (*n* = 808).

The respondent reported that their ability could be facilitated through appropriate training (74.45%), adequate knowledge (68.21%), presence of standard procedures (66.01%), patients’ acceptance of treatment (52.53%), and Suitable workplace environment for private discussion (51.75%), Figure 2.

## Discussion

The overall findings revealed that only a small proportion of the participants had adequate training in managing cognitive impairment in patients with COPD, although the majority were aware of the negative impacts associated with cognitive impairment in COPD. To the best of the authors’ knowledge, this is the first Saudi study to investigate the attitudes and physicians’ current practice of managing cognitive impairment in patients with COPD. It is important to emphasise that early recognition of cognitive impairment in patients with COPD allows appropriate intervention and, thus, improves clinical outcomes. While there is an overall agreement on the negative impacts of cognitive impairment on patients with COPD, only half of the study participants aim to recognise cognitive impairment during patients’ assessment. Further, the fact that most participants indicated not having adequate training towards managing cognitive impairment may contribute to low confidence levels in managing cognitive impairment in patients with COPD. Barriers leading to suboptimal management include the absence of standard procedures and poor training.

Current literature shows that cognitive impairment is prevalent among patients with COPD [8–10,24], with severe impacts on management and clinical outcomes [25]. Our results showed that physicians agreed on the negative impacts of cognitive impairment on patients with COPD. Indeed, cognitive impairment severely affects patients with COPD, leading to a misdiagnosis of COPD [26], under-estimation of COPD severity [27], increased dependency [4], and poor adherence to medication [3], and pulmonary rehabilitation [5]; however, a significant proportion does not see the importance of screening for the presence of cognitive impairment during patints’ visits, which may lead to a missed opportunity for optimal management. Un- or under-recognised cognitive impairment (especially at an early stage) may result in patients’ deterioration and even progression to irreversible dementia. There is indeed evidence to suggest mild cognitive impairment (MCI) – the early stage of cognitive decline - is a risk factor for developing irreversible dementia [16] and that 30% of patients with MCI may progress to dementia over a median of 5-year follow-up [16]. However, not all patients with MCI progress to dementia. It is therefore important to emphasise on the role of cognitive impairment screening/assessment [1,14] among this population.

Active screening is the first step in detecting cognitive impairment and thus starts the management process as early as possible. Indeed, assessing for cognitive dysfunction is not as complex as it sounds and can be performed using one of the validated screening tools [1], which provides an overview of whether comprehensive examinations should be conducted. Various reliable screening tools used for cognitive impairment are simple and effective [1,28]. In clinical settings, especially with limited time spent with each patient, it is important to keep in mind that screening tools should be easy, short, accurate and can assess several cognitive domains. It is worth mentioning, however, that there is no clear guidance from international COPD guidelines on how to recognise comorbidities (e.g. cognitive impairment) in patients with COPD, despite the emphasis on recognising COPD comorbidities.

Despite the increased prevalence and incidence of cognitive impairment among patients with COPD, it is hardly given any attention during patients’ assessment. Indeed, our findings showed that only half of the study participants aimed to identify possible cognitive impairment, with only a small proportion using validated screening tools. This may be explained by the fact that a significant proportion of study participants have not received adequate training, in addition to not seeing a value in screening every COPD patient. While the training curricula for internal medicine and family medicine physicians cover a broad range of medical topics, including cognitive assessment, the emphasis on cognitive symptoms in COPD is limited, leading to missed opportunities for early detection. Interestingly, pulmonologists are more likely to receive training in identifying comorbidities, including cognitive impairment. Additionally, it is possible that the time for patients’ visits is limited due to the severe shortage of healthcare workers. There is also a possibility that COPD symptoms (e.g. shortness of breath) may be pronounced during patients’s visits, and thus, be the central focus of the assessment. All of these factors, therefore, may contribute to the low detection rate of cognitive impairment in this vulnerable population.

Our findings that more than 80% of the study participants have not received adequate training for managing cognitive impairment is, in fact, alarming but may also explain why cognitive impairment remains undetected and under-reported. Indeed, evidence suggests that healthcare practitioners have poor confidence in performing cognitive assessments and; thus, are unable to detect and manage cognitively impaired patients [29,30]. Even if cognitive impairment is detected, there is the issue of uncertainty with explaining the results and taking appropriate actions. Again, unrecognised cognitive impairment has severe impacts on patients’ quality of life, functional independence, and adherence to medications and pulmonary rehabilitation. In contrast, timely detection allows appropriate intervention strategies, eventually leading to better clinical and patient outcomes.

Our findings showed that there is an overall low level of confidence when it comes to recognising, managing, and even disclosing a diagnosis of cognitive impairment in patients with COPD. Lack of training (which has been indicated by a significant proportion of participants) may translate into insufficient knowledge and skills, leading to incompetence, and, therefore, a low level of confidence. Indeed, a previous study demonstrated that nearly two-thirds of primary care physicians indicated low confidence levels when diagnosing and managing patients with dementia (without COPD) [31]. Furthermore, data from a Hungarian study also showed that GPs were unfamiliar with the concept of mild cognitive impairment, suggesting inadequate knowledge [32]. Our findings are also concordant with the current literature suggesting that there needs to be appropriate training for physicians in order to raise the level of confidence to manage cognitive impairment in patients with COPD.

Several barriers have been discussed which contribute to the suboptimal management of cognitive impairment in patients with COPD. The absence of standardised assessment, lack of time, and poor training are at the top of this list. Although timely identification of comorbidities in patients with COPD is of clinical importance, there is no clear recommendation on how to do so. There are also no validated tools that have been introduced for assessing cognitive impairment in patients with COPD, though several tools have been employed and are currently used in clinical settings. Clinicians should be aware of the strengths and limitations of each tool and how to use them. Another challenge is related to the social stigma associated with disclosing cognitive impairment (or even dementia) diagnosis to patients and their caregivers, leading to a low level of detection and suboptimal management. Patients and caregivers should be educated about the perceived benefit of early detection of cognitive impairment, as it allows timely interventions and clinical outcomes improvement.

### Strength and limitations

A major strength of this study is that it has discussed a significant gap in the current practice of managing cognitive impairment in patients with COPD. In addition, the study has recruited physicians from different disciplines across the kingdom, offering high external validity. However, this study is not without limitations. First, the nature of the study design (cross-sectional) does not allow the study of a cause-effect relationship. Instead, it only provides a snapshot of the current practice of managing cognitive impairment in patients with COPD, disregarding changes over time. Although the sampling technique used in this study may introduce selection bias, participants nevertheless were recruited from all over the country. As the physicians included in this study were from different specialities and all geographical locations in Saudi Arabia, it is less likely to introduce methodological flaws. While data collected through self-reported questionnaires may be time-efficient and cost-effective, it may introduce self-reporting bias, as physicians may overestimate their practices or confidence levels. While using closed-ended questions (as used in this study) may be helpful in quantifiable data, it may nevertheless limit gaining an in-depth understanding of the responses. Considering open-ended questions to assess physicians’ perspectives and challenges in future research may provide more insightful information. We have not distinguished physicians working in clinical offices from those practising in hospitals; the latter is more likely to diagnose more complicated COPD cases with severe comorbidities. Although it may be helpful to gain further insights from other healthcare professionals who also play a role in managing COPD patients, such as nurses and physical and respiratory therapists, this study has focused on recruiting physicians who are likely to conduct a standard assessment for patients with COPD and follow their physical illness. Thus, further studies should be performed to assess current practices and perceptions of other healthcare professionals in Saudi Arabia regarding the recognition and management of cognitive impairment in COPD patients.

### Implications of the study

Our findings imply intensive training programs for physicians in direct contact with COPD patients on screening tools for cognitive impairment, such as the Montreal Cognitive Assessment and the Mini-Mental State Exam. This will allow a clear referral path for specialised physicians (e.g. Neurologists or geriatricians) and, thus, an appropriate treatment plan. Identifying patients with COPD who are cognitively impaired also allows for taking further measures to assure treatment and appointment adherence, such as family involvement. Physicians should also be educated about the impact of cognitive impairment on patients with COPD. This will, in turn, help the physicians provide proper education to the patient’s family members (caregivers). The healthcare system should also facilitate the barriers (e.g. staff shortage, lack of time, heavy workload, and absence of standardised procedures) interfering with the optimal management of cognitive impairment in patients with COPD.

## Conclusion

Cognitive impairment is a major comorbidity among patients with COPD, contributing to underestimation of COPD severity, and poor adherence to medication and pulmonary rehabilitation, and should therefore be detected and managed as early as possible. Disappintlgy, a significant proportion of the study participants have not received adequate training, which contributes to not aiming to identify cognitive impairment during patients’ visits. Factors such as inadequate knowledge, poor training, and the absence of standardised procedures for assessing the cognitive state in patients with COPD are likely to contribute to the low detection rate. Therefore, there needs to be a holistic approach to identify cognitive impairment; and thus, intervene in order to improve clinical outcomes.

## Supplementary Material

Supplemental material.docx

## Data Availability

The data that support the findings of this study are available on request from the corresponding author, R.A.S. The data are not publicly available due to their containing information that could compromise the privacy of research participants.

## References

[1] Siraj RA. Comorbid cognitive impairment in chronic obstructive pulmonary disease (COPD): current understanding, risk factors, implications for clinical practice, and suggested interventions. Medicina (Kaunas). 2023;59(4). doi: 10.3390/medicina59040732. PMC10146750 37109690

[2] Fogg C, Meredith P, Bridges J, et al. The relationship between cognitive impairment, mortality and discharge characteristics in a large cohort of older adults with ­unscheduled admissions to an acute hospital: a retrospective observational study. Age Ageing. 2017;46(5):794–801. doi: 10.1093/ageing/afx022. 28338808 PMC5860577

[3] Allen SC, Jain M, Ragab S, et al. Acquisition and short-term retention of inhaler techniques require intact executive function in elderly subjects. Age Ageing. 2003;32(3):299–302. doi: 10.1093/ageing/32.3.299. 12720616

[4] Baird C, Lovell J, Johnson M, et al. The impact of cognitive impairment on self-management in chronic obstructive pulmonary disease: a systematic review. Respir Med. 2017;129:130–139. doi: 10.1016/j.rmed.2017.06.006. 28732820

[5] Cleutjens FAHM, Spruit MA, Ponds RWHM, et al. The impact of cognitive impairment on efficacy of pulmonary rehabilitation in patients with COPD. J Am Med Dir Assoc. 2017;18(5):420–426. doi: 10.1016/j.jamda.2016.11.016. 28108209

[6] Antonelli-Incalzi R, Corsonello A, Pedone C, et al. Drawing impairment predicts mortality in severe COPD. Chest. 2006;130(6):1687–1694. doi: 10.1378/chest.130.6.1687. 17166983

[7] Higbee DH, Dodd JW. Cognitive impairment in COPD: an often overlooked co-morbidity. Expert Rev Respir Med. 2021;15(1):9–11. doi: 10.1080/17476348.2020.1811090. 32811226

[8] Chang SS, Chen S, McAvay GJ, et al. Effect of coexisting chronic obstructive pulmonary disease and cognitive impairment on health outcomes in older adults. J Am Geriatr Soc. 2012;60(10):1839–1846. doi: 10.1111/j.1532-5415.2012.04171.x. 23035917 PMC3470752

[9] Grant I, Prigatano GP, Heaton RK, et al. Progressive neuropsychologic impairment and hypoxemia. Arch Gen Psychiatry. 1987;44(11):999–1006. doi: 10.1001/archpsyc.1987.01800230079013. 3675139

[10] Yohannes AM, Chen W, Moga AM, et al. Cognitive impairment in chronic obstructive pulmonary disease and chronic heart failure: a systematic review and meta-analysis of observational studies. J Am Med Dir Assoc. 2017;18(5):451.e1–e11. doi: 10.1016/j.jamda.2017.01.014. 28292570

[11] Siraj RA, McKeever TM, Gibson JE, et al. Risk of incident dementia and cognitive impairment in patients with chronic obstructive pulmonary disease (COPD): a large UK population-based study. Respir Med. 2021;177:106288. doi: 10.1016/j.rmed.2020.106288. 33401149

[12] Alqahtani JS. Prevalence, incidence, morbidity and mortality rates of COPD in Saudi Arabia: trends in burden of COPD from 1990 to 2019. PLoS One. 2022;17(5):e0268772. doi: 10.1371/journal.pone.0268772. 35588429 PMC9119447

[13] Chronic obstructive pulmonary disease in over 16s: diagnosis and management: National Institute for Health and Care Excellence. 2019. Available from: https://www.nice.org.uk/guidance/ng115/chapter/Recommendations 31211541

[14] Andrianopoulos V, Gloeckl R, Vogiatzis I, et al. Cognitive impairment in COPD: should cognitive evaluation be part of respiratory assessment? Breathe (Sheff). 2017;13(1):e1–e9. doi: 10.1183/20734735.001417. 29184593 PMC5702891

[15] Dodd JW, Getov SV, Jones PW. Cognitive function in COPD. Eur Respir J. 2010;35(4):913–922. doi: 10.1183/09031936.00125109. 20356988

[16] Roberts RO, Knopman DS, Mielke MM, et al. Higher risk of progression to dementia in mild cognitive impairment cases who revert to normal. Neurology. 2014;82(4):317–325. doi: 10.1212/WNL.0000000000000055. 24353333 PMC3929198

[17] Bohlken J, Jacob L, Kostev K. Progression of mild cognitive impairment to dementia in German specialist practices. Dementia (London). 2019;18(1):380–390. doi: 10.1177/1471301216673919. 27758960

[18] Farias ST, Mungas D, Reed BR, et al. Progression of mild cognitive impairment to dementia in clinic- vs community-based cohorts. Arch Neurol. 2009;66(9):1151–1157. doi: 10.1001/archneurol.2009.106. 19752306 PMC2863139

[19] Siraj RA, Alrajeh A, Aldabayan YS, et al. Attitudes, confidence, barriers and current practice of managing depression in patients with COPD in Saudi Arabia: a national cross-sectional survey. BMJ Open. 2023;13(5):e069670. doi: 10.1136/bmjopen-2022-069670. PMC10173993 37156583

[20] Siraj RA, Alrajeh AM, Alhaykan AE, et al. Assessment of the current practice of managing depression in patients with asthma in Saudi Arabia: physicians’ views. J Asthma Allergy. 2023;16:637–647. doi: 10.2147/JAA.S411614. 37384068 PMC10295812

[21] Ministry of Health. Statistical Yearbook - Health Resources. 2023. Available from: https://www.moh.gov.sa/en/Ministry/Statistics/book/Pages/default.aspx

[22] Gupta KK, Attri JP, Singh A, et al. Basic concepts for sample size calculation: critical step for any clinical trials! Saudi J Anaesth. 2016;10(3):328–331. doi: 10.4103/1658-354X.174918. 27375390 PMC4916819

[23] Kadam P, Bhalerao S. Sample size calculation. Int J Ayurveda Res. 2010;1(1):55–57. doi: 10.4103/0974-7788.59946. 20532100 PMC2876926

[24] Schou L, Østergaard B, Rasmussen LS, et al. Cognitive dysfunction in patients with chronic obstructive pulmonary disease–a systematic review. Respir Med. 2012;106(8):1071–1081. doi: 10.1016/j.rmed.2012.03.013. 22579108

[25] Roncero C, Campuzano AI, Quintano JA, et al. Cognitive status among patients with chronic obstructive pulmonary disease. Int J Chron Obstruct Pulmon Dis. 2016;11:543–551. doi: 10.2147/COPD.S100850. 27042043 PMC4801148

[26] Pezzoli L, Giardini G, Consonni S, et al. Quality of spirometric performance in older people. Age Ageing. 2003;32(1):43–46. doi: 10.1093/ageing/32.1.43. 12540347

[27] Carvalhaes-Neto N, Lorino H, Gallinari C, et al. Cognitive function and assessment of lung function in the elderly. Am J Respir Crit Care Med. 1995;152(5 Pt 1):1611–1615. doi: 10.1164/ajrccm.152.5.7582303. 7582303

[28] Cullen B, O’Neill B, Evans JJ, et al. A review of screening tests for cognitive impairment. J Neurol Neurosurg Psychiatry. 2007;78(8):790–799. doi: 10.1136/jnnp.2006.095414. 17178826 PMC2117747

[29] Bradford A, Kunik ME, Schulz P, et al. Missed and ­delayed diagnosis of dementia in primary care: prevalence and contributing factors. Alzheimer Dis Assoc Disord. 2009;23(4):306–314. doi: 10.1097/WAD.0b013e3181a6bebc. 19568149 PMC2787842

[30] Olazarán J, Torrero P, Cruz I, et al. Mild cognitive impairment and dementia in primary care: the value of medical history. Fam Pract. 2011;28(4):385–392. doi: 10.1093/fampra/cmr005. 21402661

[31] Turner S, Iliffe S, Downs M, et al. General practitioners’ knowledge, confidence and attitudes in the diagnosis and management of dementia. Age Ageing. 2004;33(5):461–467. doi: 10.1093/ageing/afh140. 15271637

[32] Imre N, Balogh R, Papp E, et al. Knowledge of general practitioners on dementia and mild cognitive impairment: a cross-sectional, questionnaire study from Hungary. Educ Gerontol. 2019;45(8):495–505. doi: 10.1080/03601277.2019.1660137.

