# Supplementary figures and images for: Managing cognitive impairment in patients with chronic obstructive pulmonary disease (COPD) in Saudi Arabia: what are the current practices?
Source: Ann Med. 2025 Jan 28;57(1):2413924. doi: 10.1080/07853890.2024.2413924 (PMC11780691; doi:10.1080/07853890.2024.2413924)

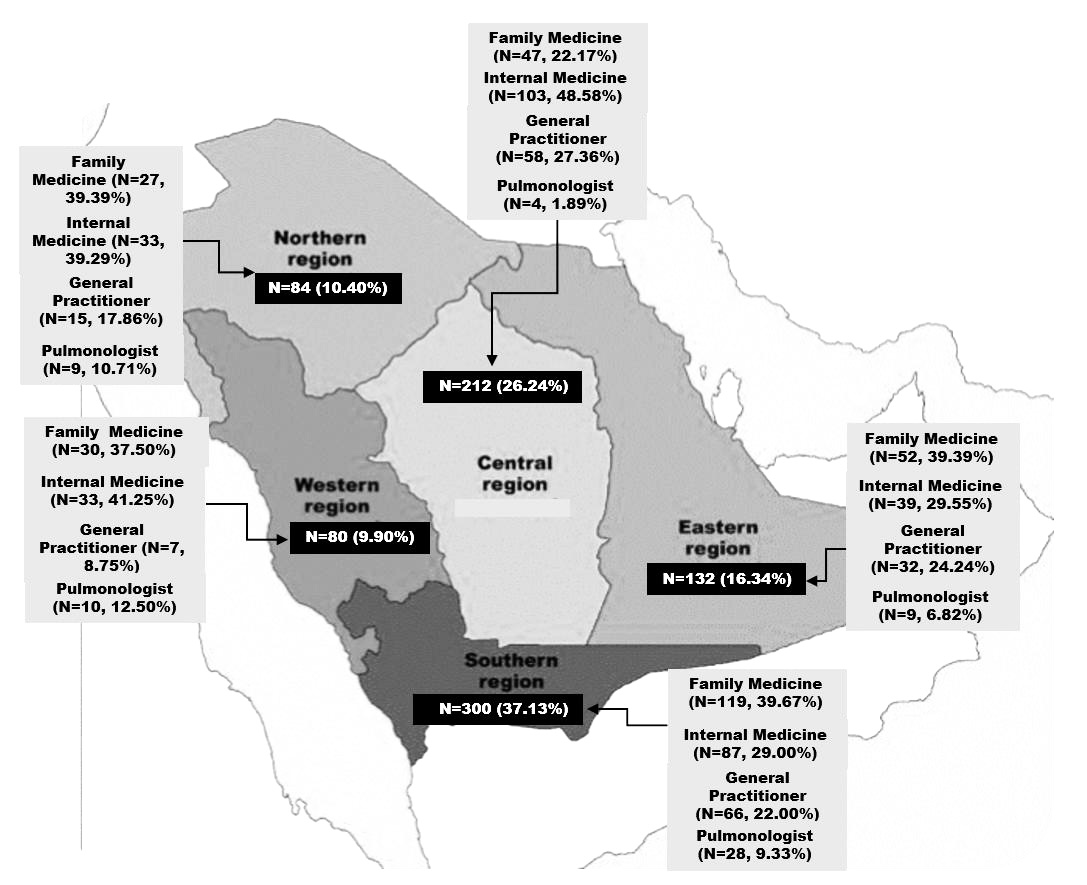


**S1.** Distribution of participants across geographical locations

Supplement: Supplemental material.docx [file IANN_A_2413924_SM6949.docx]
